# Supplementary material for: Pyruvate Dehydrogenase Kinase 1 inhibition mediated oxidative phosphorylation enhancement in cartilage promotes osteoarthritis progression
Source: BMC Musculoskelet Disord. 2023 Jul 20;24:597. doi: 10.1186/s12891-023-06585-6 (PMC10357736; doi:10.1186/s12891-023-06585-6)
Supplement: Supplementary file 1 — Additional file 1. [file 12891_2023_6585_MOESM1_ESM.zip › MF.Gsea.1653627316242/GOMF_OXIDOREDUCTASE_ACTIVITY_ACTING_ON_THE_CH_NH_GROUP_OF_DONORS.html]

Details for gene set GOMF\_OXIDOREDUCTASE\_ACTIVITY\_ACTING\_ON\_THE\_CH\_NH\_GROUP\_OF\_DONORS[GSEA]

|  || Dataset | 55457\_collapsed\_to\_symbols.cls.cls #OA\_versus\_normal.cls.cls #OA\_versus\_normal\_repos |
| Phenotype | cls.cls#OA\_versus\_normal\_repos |
| Upregulated in class | normal |
| GeneSet | GOMF\_OXIDOREDUCTASE\_ACTIVITY\_ACTING\_ON\_THE\_CH\_NH\_GROUP\_OF\_DONORS |
| Enrichment Score (ES) | -0.45820615 |
| Normalized Enrichment Score (NES) | -1.4017128 |
| Nominal p-value | 0.1 |
| FDR q-value | 0.26376858 |
| FWER p-Value | 1.0 |
Table: GSEA Results Summary

  

Fig 1: Enrichment plot: GOMF\_OXIDOREDUCTASE\_ACTIVITY\_ACTING\_ON\_THE\_CH\_NH\_GROUP\_OF\_DONORS      
 Profile of the Running ES Score & Positions of GeneSet Members on the Rank Ordered List

  

| SYMBOL | TITLE | RANK IN GENE LIST | RANK METRIC SCORE | RUNNING ES | CORE ENRICHMENT || 1 | QDPR | quinoid dihydropteridine reductase [Source:HGNC Symbol;Acc:HGNC:9752] | 112 | 0.897 | 0.1006 | No |
| 2 | BLVRB | biliverdin reductase B [Source:HGNC Symbol;Acc:HGNC:1063] | 1050 | 0.507 | 0.0897 | No |
| 3 | PYCR3 | pyrroline-5-carboxylate reductase 3 [Source:HGNC Symbol;Acc:HGNC:25846] | 2136 | 0.346 | 0.0476 | No |
| 4 | PYCR1 | pyrroline-5-carboxylate reductase 1 [Source:HGNC Symbol;Acc:HGNC:9721] | 2145 | 0.345 | 0.0890 | No |
| 5 | MTHFR | methylenetetrahydrofolate reductase [Source:HGNC Symbol;Acc:HGNC:7436] | 4334 | 0.125 | -0.0656 | No |
| 6 | DHFR | dihydrofolate reductase [Source:HGNC Symbol;Acc:HGNC:2861] | 5805 | 0.008 | -0.1787 | No |
| 7 | PIPOX | pipecolic acid and sarcosine oxidase [Source:HGNC Symbol;Acc:HGNC:17804] | 6781 | -0.061 | -0.2469 | No |
| 8 | PRODH | proline dehydrogenase 1 [Source:HGNC Symbol;Acc:HGNC:9453] | 6927 | -0.074 | -0.2492 | No |
| 9 | SARDH | sarcosine dehydrogenase [Source:HGNC Symbol;Acc:HGNC:10536] | 7211 | -0.097 | -0.2593 | No |
| 10 | PAOX | polyamine oxidase [Source:HGNC Symbol;Acc:HGNC:20837] | 7212 | -0.097 | -0.2475 | No |
| 11 | MTHFD2L | methylenetetrahydrofolate dehydrogenase (NADP+ dependent) 2 like [Source:HGNC Symbol;Acc:HGNC:31865] | 7789 | -0.141 | -0.2751 | No |
| 12 | ALDH1L1 | aldehyde dehydrogenase 1 family member L1 [Source:HGNC Symbol;Acc:HGNC:3978] | 9302 | -0.256 | -0.3613 | No |
| 13 | SMOX | spermine oxidase [Source:HGNC Symbol;Acc:HGNC:15862] | 9618 | -0.284 | -0.3511 | No |
| 14 | ETFDH | electron transfer flavoprotein dehydrogenase [Source:HGNC Symbol;Acc:HGNC:3483] | 10203 | -0.341 | -0.3548 | No |
| 15 | MTHFD1 | "methylenetetrahydrofolate dehydrogenase, cyclohydrolase and formyltetrahydrofolate synthetase 1 [Source:HGNC Symbol;Acc:HGNC:7432]" | 10249 | -0.346 | -0.3161 | No |
| 16 | ALDH4A1 | aldehyde dehydrogenase 4 family member A1 [Source:HGNC Symbol;Acc:HGNC:406] | 12080 | -0.614 | -0.3833 | Yes |
| 17 | MTHFD2 | "methylenetetrahydrofolate dehydrogenase (NADP+ dependent) 2, methenyltetrahydrofolate cyclohydrolase [Source:HGNC Symbol;Acc:HGNC:7434]" | 12239 | -0.656 | -0.3157 | Yes |
| 18 | AASS | aminoadipate-semialdehyde synthase [Source:HGNC Symbol;Acc:HGNC:17366] | 12799 | -0.968 | -0.2410 | Yes |
| 19 | CRYM | crystallin mu [Source:HGNC Symbol;Acc:HGNC:2418] | 12808 | -0.984 | -0.1216 | Yes |
| 20 | PRODH2 | proline dehydrogenase 2 [Source:HGNC Symbol;Acc:HGNC:17325] | 12847 | -1.055 | 0.0041 | Yes |
Table: GSEA details [plain text format]

  

Fig 2: GOMF\_OXIDOREDUCTASE\_ACTIVITY\_ACTING\_ON\_THE\_CH\_NH\_GROUP\_OF\_DONORS      
 Blue-Pink O' Gram in the Space of the Analyzed GeneSet

  

Fig 3: GOMF\_OXIDOREDUCTASE\_ACTIVITY\_ACTING\_ON\_THE\_CH\_NH\_GROUP\_OF\_DONORS: Random ES distribution      
 Gene set null distribution of ES for **GOMF\_OXIDOREDUCTASE\_ACTIVITY\_ACTING\_ON\_THE\_CH\_NH\_GROUP\_OF\_DONORS**
